# Progress of Syphilology

**Published:** 1874-05

**Authors:** James Nevins Hyde

**Affiliations:** Lecturer on Dermatology and Syphilis, Rush Medical College, Chicago


ARTICLE II.—*Progress of Syphilology.* By JAMES NEVINS HYDE, M.D., Lecturer on Dermatology and Syphilis, Rush Medical College, Chicago.

1. *On Syphilitic Membranoid Occlusion of the Rima Glottidis.* LOUIS ELSBERG, M.D. (American Journal of Syphil. and Dermatol.)

2. *Syphilitic Ulcerations of the Cervix Uteri.* Appendix to a translation into French of Churchill's Diseases of Women. A. LE BLOND. (Le Progres Medical, Oct. 11, 1873.)

3. *On the Treatment of Syphilis.* Prof. ZEISSL. (Wien. Med. Wochenschr.)

4. *On the Treatment of Syphilis.* A. LANCEREAUX. (La France Medic., Nov., 1873.)

5. *The Oleates of Mercury.* JOHN MARSHALL. (Medical Record, Jan. 15, 1874.)

6. *On the Question of the Transmission of Syphilitic Contagion in the Rite of Circumcision.* R. W. TAYLOR, M.D. (Reprinted from the N. Y. Medical Journal, Dec., 1873.)

1. Syphilitic membranoid occlusion of the rima glottidis requires, for its accurate determination, the use of the laryngoscope. Prof. Elsberg reports six cases treated by himself, three by Turck, one by M. Mackenzie of London, and one by Navratil of Pesth. About two and one-quarter per cent. of cases of laryngeal syphilis, are said to be complicated by occlusion.

Occurring, necessarily, in only comparatively late stages of the disease, it may be considered as a result and termination of the syphilitic process; and might be classed with tertiary syphilitic affections, were it not coincident with ulceration of the mucous surfaces, rather than of the deeper structures. In two of the eleven cases, the cartilaginous free portion of the epiglottis was reduced to a stump;* in two, its upper edge was eaten away; while in six, the cartilage was unimpaired. In no case were other cartilages involved; in two, however, the nose had fallen in.

As to the interval existing between the date of infection and the formation of the web, no satisfactory evidence was supplied by these cases. In one individual, hoarseness had existed prior to the constitutional disorder; but in none of the others was there room for suspicion of any other agent in the production of the laryngeal disease. The ages of these patients ranged from sixteen to forty-eight years; nine, however, were between twenty and thirty-three years old at the time of treatment. Nine were males, and two females, "a disproportion which we might jocularly ascribe to the proverbial constant use of the vocal bands in the latter."

The webs generally were formed of cicatricial tissue, resulting from syphilitic ulceration, and always began at the anterior angle, the greater or smaller orifice left for breathing space having been usually at the posterior extremity of the rima. In two cases, the membrane occluded the whole rima; in one, it was attached both anteriorly and posteriorly with a central orifice; in the other, it was unattached posteriorly. In still another, the membrane was adherent both in front and at the inter-arytenoid fold, but the main portion of the left vocal band was unattached, so that along its free edge there was a narrow breathing chink.

Non-membranoid laryngeal occlusion, is situated generally above the rima glottidis, and results from infiltration or swelling of the ary-epiglottic folds, laryngeal walls or ventricular spaces; cica-

* A patient with complete destruction of the epiglottis, from syphilitic ulceration, was recently exhibited to the Chicago Society of Physicians and Surgeons, by Dr. Owens, of this city. No stump, no relic of the fibro-cartilage remained. The hoarseness almost amounted to aphonia. The vocal band on one side was free, but covered by a reddened and injected membrane—that of the other, appeared to have been in part destroyed. Deglutition was almost unimpaired in a semi-recumbent position; and in no other. The case illustrated in a remarkable manner the adaptability of the neighboring organs.

tricial deformity, destruction or displacement; and neoplasms. Non-syphilitic occlusion of the rima by a membrane, may be congenital or traumatic. (Suicidal throat-cutting and surgical thyrotomy.) The nature of the obstruction, if syphilitic, is most readily determined, when there are characteristic ulcers in the neighborhood, or in the pharynx, nasal passages and skin; and when other morbid processes can be excluded.

The course of the formation of the membrane is more or less chronic, and the dyspnœa with hoarseness or aphonia, is developed so slowly that an adaptation of the organs occurs, and the symptom last described may become aggravated to a fearful degree, before becoming fatal; as in the first of these cases. Any partial occlusion is, however, highly dangerous, since it is liable to complication with superadded œdema.

General and local treatment is requisite—the former, including hygienic measures, should consist of tonics, suppletories, medicaments directed to the skin, bowels and kidneys, and iodides, if mercurialization has occurred; otherwise, the two great antisyphilitic remedies should be combined, or the mercurial administered, in accordance with Lewin's method, by the skin. For local treatment, we must be ready in every case of extreme dyspnœa, to perform tracheotomy; "*this may be needed before we can attempt to remove the web.*"

The membrane is to be removed by galvano-cautery and through the mouth. Mackenzie split the larynx open from without, in order to divide the web, and it partially re-formed. Turck operated locally in only one case, and split the web with the laryngeal knife, introduced through the mouth. Its anterior portion subsequently reunited. Navratil performed a similar operation, with like results.

Prof. Elsberg's first and second cases were not operated upon. In the third, an attempt at treatment by the galvano-cautery, failed for want of proper apparatus; the membrane was partially divided anteriorly, but subsequently reunited.

In the remaining cases, the web was successfully removed by the galvano-cautery. The instruments employed were Voltolini's (Middeldorpf's) carbon-zinc battery of two elements; his dull, knife-shaped, galvano-cautero, made in Breslau; as well as Schnitzler's, made in Vienna; and Von Brun's, made in Tubingen. The rods were surrounded with silk thread, and dipped into cold water before introduction, to prevent the conduction of heat toward their handles.

Usually a number of galvano-cauteric applications were needed, and repeated from time to time. In the fourth case, the web was entirely removed at one sitting. Little after treatment was found to be requisite beside, vocal gymnastics, the inhalation of astringent or soothing sprays, and dilatation by bougies, till the danger of reproduction of the membrane had passed.

2. Upon the cervix uteri the soft variety of chancre is oftenest found. Indurations, of rare occurrence, have however been established in cases of pescidentia. Adherent ulcers, with grayish floors, irregular borders and clean-cut edges, surrounded by a somewhat inflamed areola, are, in this location, as a rule succeeded by general infection. They may become multiple, coalescent or phagedenic. Diphtheritic and ulcerative forms are described; the latter invading the adjacent submucous tissues, and occasioning, in many cases, great loss of substance. These lesions are not generally found upon the cervical apex, as are simple ulcers, but at the point of union of the cervix and vagina. Those which are located in the cervical canal, require dilatation of the os tincæ, prior to discovery and treatment. After a variable period, their characteristic features are lost, and chancres may thus come to resemble simple ulcers, mucous condylomata, or vegetations resting upon a somewhat indurated base; the latter needing to be distinguished from cancerous degenerations. The history of such cases furnishes a clue to the diagnosis. When the surface of a simple ulcer becomes inoculated with a specific virus, it changes and becomes grayish, soft and fungous; the distinguishing features of the resulting chancre being simultaneously modified.

Mucous patches, located upon the os and cervix, are elevated and pearly-white. Their contagiousness is now generally admitted. After persistence for a longer or shorter interval, they return to the type of a simple ulcer.

3. Professor Zeissl considers that "the preparations of iodine, judiciously administered, with an appropriate regimen, are able to cause the disappearance of the first manifestations of syphilis, or, at least, to so modify the disease, that it yields to a small number of mercurial inunctions, without subsequent relapses, that is, so that the syphilis may be regarded as definitely cured."* The early syphilides, however, yielded more rapidly to a mercurial course; the later, disappearing quickly under the use of iodine. Mucous syphilides succumbed much earlier to the iodine treatment. The tincture of iodine is preferable to the iodide of potassium, not only because of its cheapness, but also on account of its inaptness for the production of the phenomena known in "iodism." Half a fluid drachm is added to six fluid ounces of water, and a tablespoonful given twice daily.

Syphilis is found to be rebellious in pregnant women infected at the time of conception, little disposed to be amenable to treatment until after confinement, and then requiring such treatment for a subsequent period.

Prof. Zeissl uses iodoform locally in indolent ulcers, and exhibits it internally in two and three grain doses, for syphilitic neuralgia—the remedy being administered in pilular form, with a bitter extract. In the event of gangrene resulting from syphilitic adenopathy, he employs the continuous water bath.

4. Lancereaux employs mercury in primary syphilis, only when there is protracted induration of the primary lesions, with well-marked adenopathy, nor does he advise cauterization; this is useless if not dangerous. When there are prodromata of an explosion of syphilis, (intense cephalalgia, great lassitude, wandering pains and moral depression)† mercury is still withheld, and,

* In an article which appeared in the July number of the *Chicago Medical Journal* for 1872, ("On Syphilitic Lesions of the Viscera,") I stated that "if it were not foreign to the subject-matter of these pages, I should proceed to show that, contrary to the advice of many syphilographers, this treatment (that by iodine compounds) can be applied to cases of primary and secondary syphilis with singular success"; and that it will "most surely and speedily procure a relief from symptoms; when the mercurial is indispensable in order to make that relief permanent." These opinions, fortified by the authority of the distinguished advocate of the doctrines of the dualistic school, cannot fail to receive the attention which they deserve.

† I have now under my care a patient who exhibited these prodromic symptoms in a marked degree, when literally on the eve of an eruption of papulo-squamous syphilides. The precedent sore had been long and extensively irritated and indurated by the caustic applications of an attending physician. Lasespecially in chloro-anæmic cases, chalybeates substituted; laxatives, if indicated by apepsia.

When the exanthemata appear, mercury is exhibited, and discontinued immediately upon their disappearance. The objections of M. Diday to such a course, in case of roseolar and papular syphilides, are not held to be valid. The administration of the metal by the mouth is preferred to the method by inunction, or Lewin's method by hypodermic injection. Where there are febrile complications, and occasionally at the outset of the disease, iodine is employed.

In tertiary syphilis, Lancereaux uses the iodide of potassium; in cases where there is visceral complication, small doses of calomel; in others, where there is room for suspicion of some lesion of the hæmatopoiœtic glands, nitric acid and mercurial inunction. Foul tertiary ulcers are dressed with glycerine, alcohol, iodine, copper, mercury, iron, iodoform, hydro-chloral and meta-chloral, (Duj. Beaumetz) and nitric acid. Arthropathy, osteocopic pain, periostites and exostoses, require vesication; the blisters being dressed with emollient cataplasms or stimulating ointments, as circumstances may indicate.

5. The oleates are clean, economical and superior to other mercurial ointments in power of penetration and diffusibility. They are absorbed by the skin with remarkable facility, and display their remedial effects with great promptitude. They are not to be rubbed in, but applied with finger or brush, lightly, in order to preclude the possibility of unnecessary irritation. In the event, however, of such an occurrence, olive oil or purified lard may be added. One grain of morphia is advantageously combined with each drachm of the oleate of mercury; the pure alkaloid is, however, to be used, since the salts of the hydro-chlorate, the acetate and the meconate are insoluble in oleic acid. From ten to thirty drops may be applied morning and night for four or five days; then at night only for an equal period, and subsequently on alternate days until a cure is effected. Salivation and constitutional irritation are not produced. Persistent and chronic inflammations, situde had existed for several days to such an extent that he reclined constantly upon a lounge—night-sweats debilitated him when asleep—gloomy forebodings added to the distress occasioned by rheumatoid pains in almost every joint. On the appearance of the eruption, these phenomena at once disappeared.

with lesions of adjacent integument, are particularly benefited by such a course.

"The oleate is to be prepared with the oxyd, precipitated by caustic potash or soda, from a solution of the metal in nitric acid, recently made and well dried. The solution of mercury by oleic acid is assisted by a temperature of three hundred degrees Fahrenheit." The five per cent. solution is a perfectly clear, pale yellow liquid, resembling, but thinner than, olive oil; the ten per cent. solution is also fluid and perfectly clear, but as dark as linseed oil; while the twenty per cent. solution is an opaque, yellowish, unctuous substance, closely resembling in appearance resin ointment. It melts very readily at the temperature of the body, and forms a kind of transparent, viscid varnish, when applied to the skin. Excessive haste and too great an elevation of temperature may reduce the metal in course of its preparation.

In cutaneous diseases the five per cent. solution without morphia is recommended, especially in tinea sycosis. In congenital syphilis a piece of the twenty per cent. solution as large as a pea or bean may be applied to the axilla night and morning for five or six days, and will produce constitutional effects without the annoyance of uncleanliness. As a topical remedy for the non-ulcerating syphilides of the head, face and neck, the ten per cent. solution is valuable as an accessory to other treatment.

6. Dr. Taylor's monograph is the result of an investigation conducted by himself at the request of the Board of Health of the city of New York, that body having had its attention directed to four cases in which it was suspected that syphilis had been communicated in the performance of circumcision.

In the first child, he discovered unmistakable lesions of syphilis upon the genital organs, and well marked, but small, papular syphilides over the trunk, arms and thighs. In the second child, the circumstances of the development of the initial lesion left room for doubt as to whether the contagion occurred in the religious rite, but did not preclude such a possibility. In the other three cases, the facts elicited, as well as analogical evidence, pointed to a local rather than to a systemic condition as the origin of the lesions of the genitals.

These children had all been circumcised by one Hebrew operator, whom Dr. Taylor had the opportunity of examining.

This man suffered from a copious eruption of tinea versicolor upon the upper portion of his trunk, and displayed a few acne-papules upon the back, but had no syphilitic history, nor had he any evidences of syphilitic disease.

Dr. Taylor concludes that (*a*) there is a possibility of the occurrence of syphilis in the Jewish rite of circumcision; (*b*) that the contagion is most likely to be communicated in the act of sucking the wound, the mouth containing a styptic liquid, or by the use of instruments soiled by syphilitic blood; (*c*) that the chances of such contagion are rendered greater by the employment of irresponsible, non-professional operators; (*d*) that the operation of sucking should be wholly abolished, and styptic solutions, if employed, should be poured upon the wound, and not squirted by the mouth; (*e*) that successive operations should not be performed without thorough and careful cleansing of all instruments; and (*f*) that physicians, or officiating rabbis, should alone be empowered to perform a rite, which, he justly adds, "has useful sanitary bearings."

The facts elicited in this investigation, induced the writer to inquire concerning the method observed by the Hebrews of Chicago, in the observation of this rite, and he was put into communication with a Jewish operator, who has for thirteen years enjoyed the almost exclusive privilege of circumcising the infants of his nationality, in this vicinity, and in several adjoining States. Strange as it may appear, his services have been in such demand that this occupation has engrossed his entire time.

It is reasonable to suppose that, with the precautions observed by him, no contagion can occur during the performance of the rite, and, in justice to him it should be added that, according to his statement, none such have occurred. The operation is very rapidly performed after drawing the prepuce through a narrow slit in a silver shield, and the styptic solution employed—alum water—is poured over the wound and never ejected from the mouth. He admits that on several occasions he has sucked the blood from the parts after the operation, when assured that there was no taint of the system, but professes to have abandoned the practice in later years. The apparatus employed is kept scrupulously clean.

